# Meath Hospital

**Published:** 1832-06

**Authors:** 


### MEATH HOSPITAL.

*Clinical Observations on the Exhibition of Opium in large Doses in certain Cases of Disease.* By Wm. Stokes, m.d., one of the Physicians to the Meath Hospital. (Condensed from the *Dublin Journal of Medical and Chemical Science*, No. 2.)

Dr. Stokes is of opinion that practitioners do not sufficiently estimate the powers of opium in subduing inflammatory action, and the principal object of his paper is to establish this point.

*In Peritonitis occurring under circumstances where bloodletting cannot be employed,* opium appears peculiarly advantageous.

" 1st. Peritonitis arising from the escape of fæcal matters into the peritoneal cavity, through a perforating ulcer of the intestine.

" 2d. Peritonitis arising from the bursting of an abscess into the serous cavity.

" 3d. Peritonitis occurring after the operation of paracentesis in debilitated subjects.

" In addition to these cases which I have myself witnessed, we may add, that low typhoid peritonitis, occurring after delivery, as described by Drs. Cusack and Gooch; and the peritonitis which results from rupture of the intestine, induced by external violence."

The first of these forms of peritonitis has been accurately described by Louis, in his Anatomico-Pathological Researches. Many examples of this disease have been seen in the Meath Hospital, in which, with scarcely an exception, an accurate diagnosis was made. The diagnosis in these cases is generally not difficult, being principally founded on two circumstances:

"1st. The sudden supervention of the peritonitis.

"2d. The rapid sinking of the powers of life.

"These circumstances occurring in a case where there were previous indications of a diseased state of the intestinal mucous system, formed the ground of our diagnosis. I need scarcely add, that we owe this to the researches of Louis.

"There is here a sudden solution of continuity, and the diagnosis is founded on the same principles as that of other internal ruptures; for example, in cerebral apoplexy, rupture of the heart, or of aortic aneurism, pneumo-thorax from fistula, rupture of the bladder and uterus. It is true that some of these accidents may take place without this suddenness of symptoms, so will also the disease under consideration; but these are exceptions to a general rule."

"We cannot bleed in these cases, for the powers of life sink almost instantaneously. We cannot use mercury internally, for any thing that excites the peristaltic action of the intestine will of course tend to keep open the communication, and the external use of mercury is too slow in its action to arrest the disease. The means of treatment, then, in common peritonitis, are unavailable, a doctrine quite consonant with theory, and receiving support from melancholy experience."

In the treatment of this disease there are two indications: to support the strength of the patient, as far as this can be done without injury; to prevent the further effusion in the peritoneal cavity, by endeavouring to induce organization and adhesions of the effused lymph. It is obvious that, towards fulfilling this last indication, all that is wanted is time, and to diminish the peristaltic action of the intestine as far as possible; and Dr. Stokes is of opinion that, in the exhibition of opium in large doses, we have a remedy calculated to fulfil these ends. The following cases are related:

"A boy, aged twelve, was admitted into the Meath Hospital, in September 1829. He had been ill for ten days with symptoms of an inflammatory affection of the digestive tube, for which no proper treatment had been adopted for the first seven days. He was then seen by Mr. Pakenham, on account of a sudden aggravation of symptoms. This gentleman found him in a state of great exhaustion; pulse fifty-eight, sharp and intermittent; countenance sunk; the belly universally tender; frequent vomiting; diarrhœa and tenesmus. The belly was leeched, and calomel and opium exhibited. Next day, the pulse having risen, a small bleeding was performed, but the blood was not inflammatory.

"On admission, his countenance was collapsed, anxious, and expressive of dreadful suffering, the extremities were cold, and the pulse hardly perceptible. The respiration hurried, and the abdomen swelled, and exquisitely painful.

"In this case I made the diagnosis of peritonitis, from ulcerative perforation of the intestine. It was plain that the antiphlogistic treatment could be pursued no further. From having witnessed the good effects of the opium treatment in the case of peritonitis, from purulent effusion, under Dr. Graves, I determined on attempting the same treatment. The black drop was given every second hour, so that in the next twenty-four hours the patient had taken sixty drops of this preparation. I also directed mercurial frictions to the belly, but these seemed to have no influence on the system. Next day, the most marked improvement had taken place; the pulse had become full and soft, and the extremities warm; the countenance had altogether lost the hippocratic expression, and the patient could bear pressure on the abdomen. On the day before, he was nearly insensible to surrounding objects, but now expressed great relief, and confidence in recovery. The same treatment was persevered in for the next twenty-four hours, when all symptoms of abdominal inflammation had completely subsided. The belly felt natural; there was no tenderness; the pulse was good, and the patient declared himself well. At this period of the case I omitted the opium, and exhibited the mildest possible saline laxative, as no stool had taken place for more than forty-eight hours: four evacuations took place, followed by an immediate return of all his former symptoms, under which he speedily sunk.

"*Dissection.* The abdomen was distended, and tympanitic; the intestines were every where agglutinated together, and adherent to the parietal peritoneum, except in the left iliac fossa, where a quantity of yellow puriform matter was collected. Small quantities of the same fluid were found between several of the intestinal folds, circumscribed by the effusion of lymph; general livid redness of the serous membrane. On detaching the caput coli from the peritoneum lining thc right iliac fossa, a small perforation of the gut was discovered, by the escape of the contents of the intestine in a jet. The serous membrane lining the iliac fossa was found of a bluish colour, and softened for a space of three square inches; this change was also observed in the lower portion of the ileum. The perforation of the large intestine before mentioned was apparently the result of an aphthous ulceration, as no glandular enlargement, or ulceration, could be detected round it. There, however, the mucous membrane was softened, and of a deep red colour, while the remaining portion of the intestine was healthy.—*Dub. Hos. Reports*, vol. v.

"I shall remark merely on this case, that, if my mind had not been warped by an early and unfounded prejudice as to the necessity of evacuations from the bowels, the life of this patient would in all probability have been saved. It is plain, that if in any case purgative medicine is more hazardous than in another, it must be here, where the peristaltic action will constantly tend to prevent the closing of the ulcerated communication. The slight extent of disease in the mucous membrane increases the probability that under other circumstances this patient would have recovered.

"The next case had a more fortunate result, which I attribute to the dear-bought experience of the last.

"A middle-aged man was admitted on the 27th of June, 1830, apparently in the last stage of peritoneal inflammation. The disease was of three days' standing, had supervened suddenly, in a few days after hypercatharsis, induced by a large dose of glauber salts, and followed by long-continued exposure to cold. It was attended by the usual symptoms of peritonitis, from ulcerative perforation of the intestine. The belly was swollen, and so exquisitely tender, that the slightest pressure made the patient utter screams. The countenance was hippocratic, and the patient tormented with constant hiccup. Coldness of the extremities had commenced, and the pulse was weak and slow. Before the hour of visit, leeches had been applied to the belly without relief, the patient was then ordered one grain of opium every hour.

"The next day, it was found that the symptoms had improved. The patient had not experienced the slightest coma, headach, or delirium. The same plan of treatment was persevered in, the daily dose of opium being gradually diminished until the 7th of July, when the convalescence being completely established, the remedy was omitted. During this time diarrhœa set in for three or four days severely: this was treated by the application of a few leeches to the anus, and the use of anodyne enemata.

"The patient took in all one hundred and five grains of opium, (exclusive of that in the injections) without ever experiencing any of the usual effects of this substance, when exhibited in large doses. —*See Mr. Hart's Essay, Dublin Hospital Reports,* vol. v.

"I would submit, that in this case there could be little doubt but that the peritonitis arose from an ulcerative perforation of the intestine. The symptoms were perfectly similar to those in which we had before unerringly made the diagnosis, and the supervention of the peritonitic symptoms after the hypercatharsis is quite in accordance with this view. The power of bearing these great doses of opium in these cases is most remarkable, and is almost always observed. How far the exemption from its usual effects on the nervous system is explicable by the violence of the abdominal disease, is worthy of consideration."

Since the occurrence of these cases, Dr. S. has used the same remedy in instances of ordinary peritonitis, where bleeding was inadmissible, and he has had no reason to change his high opinion of its powers. He would also propose it as a remedy in cases of rupture of the bladder and uterus, and in peritonitis after the operation for strangulated hernia. In two cases of peritonitis after tapping, where the patients were in a low state before the operation, the exhibition of these large doses of opium, without general or local bloodletting, has succeeded, in his practice, in saving the life of the patient.

"It was in a case of this kind which occurred in the old Meath Hospital, in the year 1822, that Dr. Graves first ascertained the great importance of opium in this disease. A woman had laboured nnder ascites, for which she was tapped; the operation was followed by the symptoms of peritonitis. When Dr. Graves saw her, she appeared in the last stage of the disease; she had constant vomiting, hippocratic countenance, cold extremities, the belly exquisitely tender, and the pulse 160 in the minute, and nearly imperceptible. The case appearing hopeless, Dr. Graves determined on merely endeavouring to allay the distressing symptom of vomiting, and administered a drachm of laudanum. The patient soon after fell asleep, and awoke refreshed, with a more warm surface and fuller pulse; the vomiting had ceased. The same remedy was used in smaller doses every fourth hour, and in the course of two days all the unpleasant symptoms had disappeared."

Dr. Stokes also submits some cases of diseases of mucous membranes, in which the same treatment proved efficacious. The first is that of a gentleman, who laboured under all the symptoms of gastro-enteritis in a severe form.

"He had considerable fever; thirst urgent; constant smacking of the lips; respiration hurried, without disease of the respiratory system; a red tongue, and great tenderness of the belly. The usual treatment was pursued, but the disease shewed great obstinacy, and after six weeks' continuance the situation of the patient appeared hopeless. At this time a violent bronchitis supervened, and so great were the sufferings of the patient from the accumulation of mucus in the trachea, that on three occasions I left him, never expecting to see him again. It was remarkable that, during this attack, the symptoms of abdominal disease greatly subsided. Under a stimulating treatment he recovered from the bronchitis, only to relapse into his former state. The abdominal symptoms now became still more urgent; the belly swelled from tympanitis; the verge of the anus became surrounded by large and irritable hæmorrhoids; there was extraordinary prostration and constant low delirium. Under these circumstances, a diarrhœa supervened, at first slight, but afterwards so severe as to threaten death every day from exhaustion. A great variety of means were tried, but without avail. At this time, when the patient seemed in articulo mortis, I ordered him a grain of opium every hour; this he took regularly for the first twelve hours, without any inconvenience, and he experienced some refreshing sleep. Next day, the remedy was continued in the same dose every second hour, and from this time his improvement was rapid; and I rejoice to say, that he is now in the enjoyment of good health.

"This was a fair case for the trial of this heroic remedy, as all our other means had failed. I have never seen a disease more intractable to common treatment than the diarrhœa under which this gentleman laboured.

"The next case is one to which I confess I look back with great pleasure. It is that of a patient named Molyneux, who was admitted in the beginning of February last into the Meath Hospital, complaining of sore throat and pain shooting through both ears. His countenance was haggard; his voice raucous; and the body emaciated. An extensive and unhealthy looking ulcer, covered with a whitish matter, was found to occupy the left tonsil, the back of the pharynx, and left side of the uvula. The patient denied having had syphilis, but circumstances led us to suspect this; he had, however, been frequently salivated in India for abdominal disease and fevers. He first felt the soreness of his throat six weeks before admission, which was the time when his vessel made the British Channel. We ordered him the sarsaparilla decoction with nitro-muriatic acid, and touched the sore with a strong solution of nitrate of silver, which caustic was changed in some days for the butter of antimony. No good effect was produced by these means, the sore extended quite round the uvula, which it rapidly destroyed. The breath became fœtid; the cough laryngeal; the patient's appearance was still worse than on admission; his nights were sleepless, and he complained much of pain in the head.

"I now changed the plan of treatment; omitted the sarsaparilla and the lotion, and ordered a gargle of chloride of lime, with the internal use of six grains of opium daily, and an increase of his wine. At once the sore began to assume a more healthy appearance; the fœtor of breath diminished greatly, and in a few days wholly disappeared. After a short time, in consequence of want of sleep, we increased the dose to eight grains, on which he has been kept since the 20th of February. The sore is now healed, and the whole state of the patient singularly improved.

"This man has, in the course of a few days, taken upwards of a hundred grains of opium, without experiencing any of its poisonous effects. His slumbers have been light and interrupted, his intellects clear, and his bowels have not been constipated, as he has had one evacuation daily since the beginning of this treatment. He only complains of a slight difficulty in passing urine. In this respect, the case resembles those which I have brought forward, and I may here mention, that whenever the remedy produces its poisonous effects, it appears to be a sign in that particular case, its singular influence over inflammatory action will not be produced. Like the tartar emetic, as observed by Laennec, it acts best where there is no sensible effect produced, but the diminution of the local disease.* There is at present in the Meath Hospital a woman, who for an enteritis has taken eighteen grains of opium in the last

* "It is most interesting that, in this case and in another, no narcotism was produced until the patient was convalescent, as if the existence of the disease caused the tolerance of the opium."

forty-eight hours: in this case there was, previous to the use of opium, retention of urine, but this has now completely subsided."

From the facts he has recorded Dr. S. deems the following conclusions justifiable.

"1st. That in certain cases of inflammation of serous and mucous membranes, where depletion by bloodletting, or other antiphlogistic measures, are inadmissible, and the system in a state of collapse, the exhibition of opium has a powerful effect in controlling the disease.

"2d. That under these circumstances the remedy may be given in very large doses, with great benefit and safety.

"3d. That its effect then is to raise the powers of life, and remove the local disease.

"4th. That the poisonous effects of opium are rarely observed in these cases; the collapse and debility of the patient appearing to cause a tolerance of the remedy.

"5th. The cases in which the utility of this practice has been ascertained are as follows:

"Simple peritonitis, in a stage where bleeding cannot be performed. Low puerperal peritonitis. Peritonitis from perforation of the intestine; from the opening of an abscess into the sac; or lastly, after the operation of paracentesis in debilitated subjects. Violent diarrhœa, supervening in exhausted subjects. Phagedenic ulceration of the throat, in similar individuals. And cases of chronic gastritis, and gastro-duodenitis in patients exhausted by the long continuance of the disease.

"6th. The cases in which this mode of treatment would be probably useful are, peritonitis from rupture of the bladder, or uterus, traumatic rupture of the intestine, or after the operation for strangulated hernia.

"The last observation which I shall make here is, that in most of these cases, particularly in those of diseases of serous membranes, wine was given in conjunction with the opium, and in all the patients were supported by a lightly nutritious diet."

